# OBESITY AND ACCELERATED AGING

**DOI:** 10.1093/geroni/igae098.0323

**Published:** 2024-12-31

**Authors:** Dennis Villareal

**Affiliations:** Baylor College of Medicine, Houston, Texas, United States

## Abstract

Obesity in the older population has increased in prevalence and incidence over the last several decades and has been associated with a decline in physical function, quality of life, and increased risk of complications from cardiometabolic diseases. Several interrelated hallmarks of aging have been proposed. The pathophysiologic changes that occur with obesity are similar or contribute to those that occur with aging. In fact, the positive energy balance typical of obesity worsens the excess deposition of ectopic fat with aging. Obesity and aging are characterized by chronic low-grade inflammation, which contributes to reduced muscle quality and protein control mechanisms. Obesity causes oxidative stress and inflammation, which can increase telomere shortening. Obesity induces epigenetic alteration, which can accelerate age-related dysfunction. Calorie excess increases formation of reactive oxygen species (ROS), which damages nucleus, endoplasmic reticulum, and mitochondria. DNA damage from ROS upregulates p16 and p21, resulting in chromatic reorganization, cellular senescence, and production of proinflammatory factors (senescence-associated secretory phenotypes [SASP]). Persistent DNA damage associated with inefficient DNA repair with both obesity and aging may be the principal molecular event that underlies accelerated aging. Both obesity and aging deregulate cellular mechanisms related to nutrient signaling. Caloric restriction in combination with exercise slows biologic aging by protecting against the molecular and cellular damages that occur in obesity and aging.

